# EU health information progress: the harvest of policy supporting projects and networks

**DOI:** 10.1186/s13690-021-00772-4

**Published:** 2022-01-30

**Authors:** Mariken J. Tijhuis, Linda A. Abboud, Peter W. Achterberg

**Affiliations:** 1 National Institute for Public Health and the Environment (RIVM), Bilthoven, The Netherlands; 2 Sciensano, Brussels, Belgium

**Keywords:** Health information, International comparison, Health policy, Population health monitoring, Health system performance, Networks, Projects, Infrastructure, European Union

## Abstract

**Background:**

The European Commission supports the initiation of health information related projects and networks serving comparative population health monitoring and health system performance assessment. Many of these projects and networks have produced relevant data, standards, methods, indicators and knowledge that may be lost as these networks become inactive.

The aim of this project retrieval and review was to identify health information projects and networks and their produced output; and subsequently facilitate systematic access to this information for policy makers, researchers and interested others via a web-based repository.

**Methods:**

The scope of this article covers 1. population health oriented topics and 2. health system/health services oriented topics. Out of scope are specific infectious diseases; individual rare diseases; and the occurrence and effects of specific medical treatments, interventions and diagnostics; cohort studies; or studies focusing on research methods.

We searched bibliographic databases and EU project databases for policy supporting projects and networks and selected those fulfilling our inclusion criteria after more in-depth inspection. We searched for their outputs. In addition, we reviewed country participation in these projects and networks.

**Results:**

We identified 36 projects and networks, 16 of which are population health oriented, 6 are health systems and services oriented and 14 cover both. Their total volume of output is not easily retrievable, as many project websites have been discontinued. Some networks and/or their outputs have found continuance within European agencies and/or national institutions. Others are struggling or have gone lost, despite their policy relevance. Participation in the projects was not evenly distributed across Europe. Project information was made available through the Health Information Portal.

**Conclusions:**

EU funded projects and networks have contributed greatly to the evidence-base for policy by providing comparative health information. However, more action is needed to evaluate and conserve their outputs and facilitate continued contribution to the field after project funding stops. The realization of a sustainable infrastructure for these projects and networks is urgent. The Health Information Portal can play an important role in conserving and reusing health information. Information inequalities may exist across Europe but need further investigating.

**Supplementary Information:**

The online version contains supplementary material available at 10.1186/s13690-021-00772-4.

## Background

A solid base of European comparative health data and indicators is essential for European Union (EU) Member States’ (MS) ability to conduct comparative analyses, benchmark against other countries or international targets, monitor progress, and develop and evaluate evidence-informed policies. This requires a solid routine data collection and analysis mechanism such as available at the European Union Statistical Office (Eurostat), World Health Organisation (WHO) and Organisation for Economic Collaboration and Development (OECD), complemented with data and indicators from other sources. Attempts to improve international comparability of data and indicators often run into issues of national and international interoperability, and challenges for reuse of data for quality assessment, benchmarking, monitoring and system assessment and research purposes.

The importance of attaining a solid health information base was first addressed by the European Commission (EC) in 1997 through its initiative for a Health Monitoring Programme (HMP, 1997–2002) [1]. This programme intended to pave the way for permanent EU health monitoring and (co) funded projects that collected data, harmonized data collections, developed health indicators, and produced articles and reports that supported the evidence and information base for national and international health policy making. Thereafter, consecutive public health programmes, consisting of 3 multiannual health programmes (2003–2008; 2008–2013; 2014–2020) continued to (co) support health information projects and expert networks. In addition, framework programmes, stimulating research in the European Research Era, also aimed to contribute to the development and collection of comparative data and affiliated research networks. Another support mechanism is that of Cost Action, where the focus is on connecting researchers in specific domains.

Yet, the sustainability of these networks and their project results is not self-evident. Once funding ends, projects are not systematically followed-up. This is visible, for example, from the lack of maintenance of the websites that host the generated content and knowledge built within these projects and networks. This challenge is inherent to any project-based funding system. However, this also points at the difficulty for international networks to coordinate their data collections, knowledge and participants and to attain or keep full EU MS coverage and commitment, even when results are relevant. Also, it holds the danger of having to reinvent the wheel after some years as existing relevant information is lost or difficult to retrieve.

A goal of the current work was to provide users of health information with a (non-exhaustive) repository of EU subsidized comparative health information projects and networks that contribute to policy support. In this, we aimed at efforts in population health monitoring, health system performance assessment and national and international health information systems in general. The scope and terminology are clarified in Box 1. Simultaneously, we aimed to explore if large differences exist in the participation rate of MS in these networks and projects, as this could possibly indicate health information inequalities among MS. This may be an important obstacle to a well-functioning European health information system. Inequality in health information across Europe is a problem in itself, as it leads to underestimates of the health inequalities described [5]. 
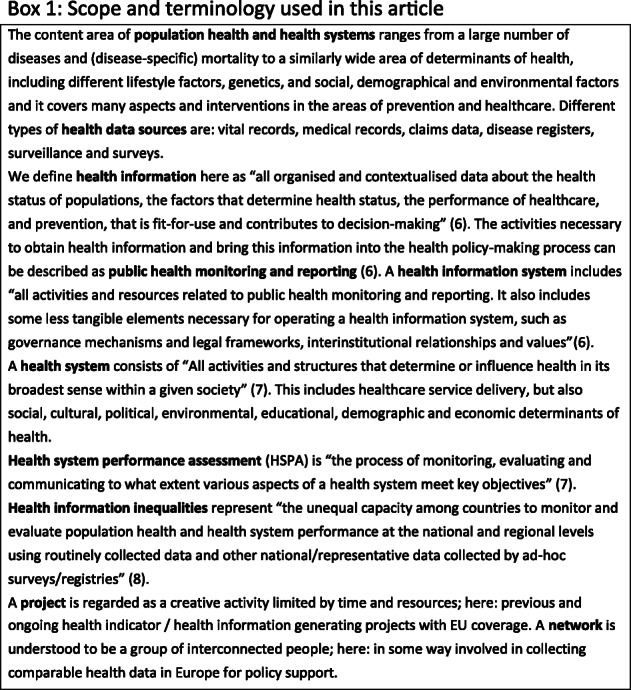


The work is conducted within the Joint Action on Health Information (InfAct, Information for Action!, 2018–2021) [6]. InfAct aimed to build towards a sustainable and solid infrastructure for EU health information and strengthen its core elements based on capacity building, health information tools and political support. One of the goals of InfAct was to bring together existing international networks, data and expertise to strengthen their efforts and enable new and emerging other networks to learn from their best practices and provide opportunities for capacity building. InfAct developed the ‘European Health Information Portal’ [7], which is currently continued under the Population Health Information Research Infrastructure (PHIRI) project [8].

In short, this article aims to provide more insight in European health information projects and networks that support evidence-informed policies and explore potential information inequalities therein.

## Methods

### Selection of projects and networks: sources, strings, limits and in/exclusion criteria

The focus of our inventory is on those projects and networks, (co-)funded by the EC, that contribute to policy support by strengthening 1) population health monitoring and 2) health system performance assessment by improving and/or providing internationally comparative data that are not (necessarily) produced as routine statistics, datasets and surveys by the international organisations such as Eurostat, WHO and OECD.

We searched PubMed, Embase, Scopus, Google, the CHAFEA health programmes database [9], the Cordis database [10] and the ECHI metadata [11]. These sources were followed up by hand-search. The COST action website has a browse function [12], but was not part of our search strategy.

The search was performed simultaneously for both topics in our scope and we prepared strings for the following terms:

A. network, data collection.
B. project, data collection.
C. public health, monitoring, health system, health care, performance.

The above-mentioned sources were searched from 2010 onwards to keep the results manageable and not overly outdated. An exception to the date limit was made for the “Health Information” calls 2004–2007 in the CHAFEA project database and the ECHI metadata. The search was limited to EU MS and associated countries’ networks and projects, in the English language. The search terms are provided in Additional file 1.

PubMed, Embase, Scopus and Google were first searched by an information specialist from March through May 2019 and then further screened by members of the InfAct project team. The results were collected in an Endnote file. A total of 1575 publications were identified in the first screening round. Of these, 336 were selected for further in-depth screening.

We searched the Cordis database and the CHAFEA health programmes database in July 2019 and updated the search on April 15th, 2020. The results (*n* = 765) were collected in an excel file for further screening, first based on objective, then in a more in-depth manner.

The inclusion and exclusion criteria applied to reach the final selection are shown in Table 1. The reuse of comparable health data, harmonised indicator development and their use for health policy support is the core of the selection. This selection also serves as the basis for the analysis of MS’ participation in the projects and networks as well as for the estimation of their scientific impact in terms of producing scientific articles and reports.

**Table 1:** Inclusion and exclusion criteria for public health and care networks and projects

	Inclusion	Exclusion
Scope	Public health Population health Health care performance Health systems performance	Infectious diseases Individual rare diseases
Activities	Data/information-collecting; Strengthening MS and/or EU health information systems and/or facilitate the use of evidence-based health information for health policy making	Non-data/information collecting, NGO type networks, commercial networks, networks on legal, ethical and governance issues, or networks in scientific areas where health is a side topic, epidemiological (cohort) analyses that are not used for MS comparisons
Data collection (where applicable)	Longitudinal, periodic collection	One-time cross-sectional study
Outcomes	Health related outcome and performance measures	Occurrence and effects of individual treatments, interventions and diagnostics; best practices in clinical settings
Geographic coverage	EU and associated countries	other
Geographic coverage	≥ 5 countries	< 5 countries
Funding	Health Program, Framework Program, Cost Action, other	Non-EU

Some networks and projects were successors of previous ones, under the same or a different name. These were clustered as far as we could identify them.

### Extraction of information

For each of the identified projects and networks, we collected the following information: website (if still available), project site in EU database, funding program, start and end date, project aim, topic orientation (population health/health systems), number of outputs in EU database, number of outputs in PubMed, leading Institute, and participating countries (for which we registered EU and European Free Trade Association (EFTA) countries).

We divided the projects and networks based on their orientation towards population health or health systems/services. If projects/networks cover both, then they were classified as such.

#### Outputs

As a rough estimate of scientific impact, we searched PubMed for the project name and collected this in an Endnote file. We checked for duplicates in case of related projects, but did not attempt to take out errata, or publications that mentioned the project but were not produced by it. In case of several projects succeeding each other, we used the sum of the articles. We also searched for other types of outputs, such as reports, in the projects databases and websites, and sought to quantify them.

#### Project participation

We assessed the identified projects for country participation (EU MS and European Free Trade Association countries) via the EU-database or project website. In case of multiple projects linked to each other we used the participating countries in the most recent project.

## Results

Our search and selection resulted in a wide array of projects, covering the domains of health determinants, health status, health services, health promotion and contextual issues. Additionally, the projects covered a variety of topics, including child and perinatal health, elderly health, chronic diseases (most notably cancer, diabetes, cardiovascular disease), environmental health and injuries. Table 2 lists the projects, the date of the index project and related/previous projects. These projects, and more, are also listed on the Health Information Portal.

**Table 2:** list of index projects, their time frame and their link to previous/other projects

Acronym	Full name	Time frame	Embedding and project links
**AMIEHS**	Avoidable mortality in the European Union: towards better indicators for the effectiveness of health systems	2008–2011	
**BRIDGE Health**	BRidging Information and Data Generation for Evidence-based Health Policy and Research	2015–2017	Opportunity for ECHI, EHES, EUBIROD, EU-IBD/EuroSafe, EHLEIS, EUROCISS, EuroHOPE, Euro-Peristat, EUROREACH and RICHE to work on sustainable future for (net)work.
**CHILD**	Child health indicators of life and development Project	2000–2002	
**CHRODIS PLUS JA**	Implementing good practices for chronic diseases	2017–2020	CHRODIS JA (2014–2017)
**EATWELL**	Interventions to Promote Healthy Eating Habits: Evaluation and Recommendations	2009–2013	
**EBoDN**	European Burden of Disease Network	2019–2023	
**EHES**	European Health Examination Survey	2009–2012	FEHES (2006–2008)
**ENHIS2**	Establishment Of Environmental Health Information System Supporting Policy Making	2005–2007	
**EUBIROD**	EUropean Best Information through Regional Outcomes in Diabetes	2008–2012	BIRO (2005–2008)
**EUCID**	EUropean Core Indicators in Diabetes Mellitus	2006–2008	
**EUnetHTA**	European Network for Health Technology Assessment JA3	2016–2021	EUnetHTA (2006–2009), EUnetHTA JA (2010–2013), EUnetHTA JA 2 (2012–2016)
**EUNICE**	European Network for Indicators on Cancer	2006–2008	
**EUPHID**	European Health Promotion Indicator Development Project	2001–2004	
**EuroCARE**	EUROpean Cancer Registry-based study	1998–2001	Eurocare 1 (1990–1992); Eurocare 2 (1995–1998)
**EUROCAT**	Registry of Congenital Anomalies	2011–2014	EUROCAT (2004–2007); EUROCAT (2007–2010)
**EUROCHIP**	European Cancer Health Indicator Project	2008–2012	EUROCHIP (2004–2008)
**EUROCISS**	European Cardiovascular Indicators Surveillance Set	2004–2007	EUROCISS (2000–2003)
**EuroCoDe**	European Collaboration on Dementia	2006–2009	
**Euro-DEN**	European drug emergencies network	2013–2015	
**EURO-HEALTHY**	Shaping EUROpean policies to promote HEALTH equitY	2015–2017	
**EuroHOPE**	European Health Care Outcomes, Performance and Efficiency	2010–2014	
**EUROMOMO**	European monitoring of excess mortality for public health action	2008–2011	
**Euro-Peristat**	Better Statistics for Better Health for Mothers and their Newborns in Europe	2011–2014	Euro-Peristat 2 (2004–2007); Euro-Peristat III (2008–2010)
**EUROREACH**	EuroREACH A Handbook to Access Health Care Data for Cross-country Comparisons of Efficiency and Quality	2010–2013	Health Data Navigator
**EUROTHINE**	Tackling Health Inequalities in Europe	2004–2007	
**HLS-EU**	The European Health Literacy Survey	2009–2012	
**I2SARE**	Indicateurs d’inégalités de santé dans les régions d’Europe; Health inequalities indicators in the region of Europe	2008–2010	ISARE (1999–2001)
**INTEGRIS**	Integration of European Injury Statistics	2008–2011	
**JA ECHIM**	European Core Health Indicators	2009–2013 (1998–2013)	ECHI 1 (1998–2001), ECHI 2 (2002–2005), ECHIM (2005–2008); Projects in this table related to development of the indicators are: I2SARE, PROMeTHEUS, EUBIROD, CHILD, EHEMU/EHLEIS, EHES, ENHIS2, EUCID, EuPHID, EuroCARE, EUROCHIP, EUROCISS, EuroCoDe, Euro-Peristat, IDB
**JA EHLEIS**	The joint action on healthy life years	2011–2014	EHEMU (European Health Expectancy Monitoring Unit, 2004–2007); EHLEIS (European Health and Life Expectancy Information System, 2007–2010); HEX.net (Health Expectancy Network, 2021)
**JAMIE**	Joint Action on Monitoring Injuries in Europe	2011–2014	European Injury Database; EUROSAFE
**MOCHA**	Models of Child Health Appraised	2015–2018	
**PROMeTHEUS**	Health Professional Mobility in the European Union Study	2009–2012	
**REITOX**	European information network on drugs and drug addiction (Réseau Européen d’Information sur les Drogues et les Toxicomanies)	Started 1992	
**RICHE**	A platform and inventory for child health research in Europe	2010–2013	
**SHARE**	Survey of Health, Ageing and Retirement in Europe	2002–2004	As of 2011, SHARE is a European Research Infrastructure Consortium (ERIC)

Our final selection consisted of 36 projects and networks, in total. Of these, 16 are population health oriented (PH) and 6 are health systems and services oriented (HSS) and 14 consider both (see also Table 3). Most projects have been co-funded by the DG SANTE Health Programme. The projects are equally spread across the past 15 to 20 years, however many projects shown have predecessors, of which some go back longer than 20 years (see Table 2).

**Table 3:** Project description in numbers

	All	PH	HSS	Both
**Projects**	36	16	6	14
**Funding**				
**Health (Monitoring) Programme**	23	10	2	11
**Framework Programme**	10	3	4	3
**Other**	3	3	0	0

*PH* population health, *HSS* health systems/services

### Outputs

The number of retrieved research output using PubMed ranged from 0 to > 500. The median number for the public health (PH) oriented networks was 7 (mean: 31) and for the health systems/services (HSS) this was 10 (mean: 92).

It was not possible to quantify the outputs in terms of reports, although this might be more relevant for policy support.

### Project participation

**Fig. 1:**
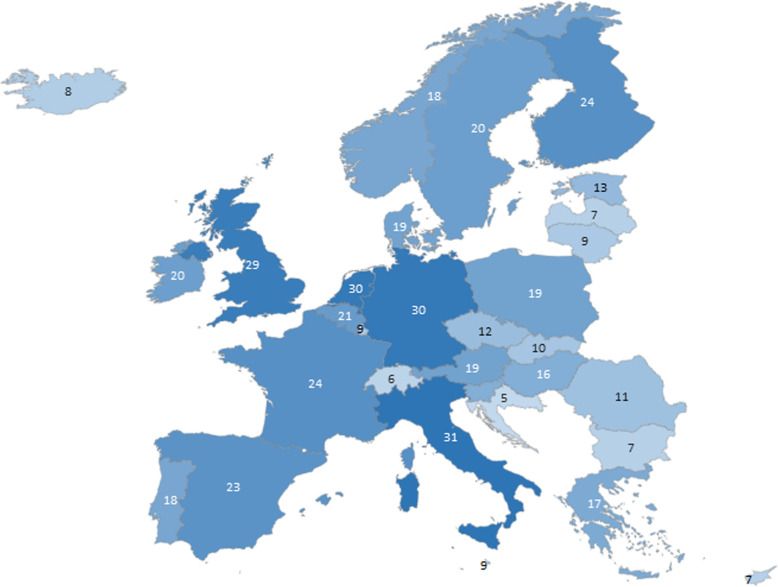
Geographical presentation of participation in selected networks & projects. numbers represent the number of projects a country participated in

The most active network & project participants were Italy, the Netherlands, Germany and the UK (see Fig. 1). It must be noted that the collected numbers refer to active participation in EU-(co) funded projects and networks, via one index-project. Eastern EU countries appear to participate the least.

**Fig. 2:**
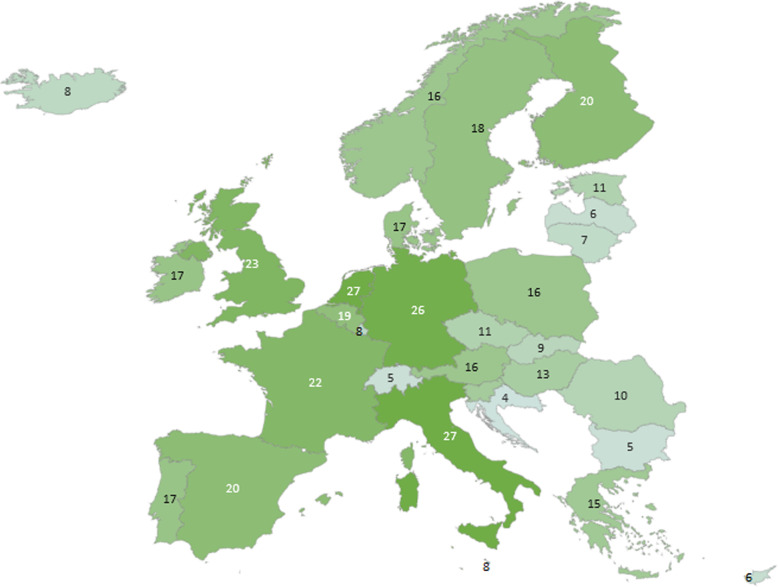
Geographical presentation of participation in selected PH networks & projects. numbers represent the number of projects a country participated in

**Fig. 3:**
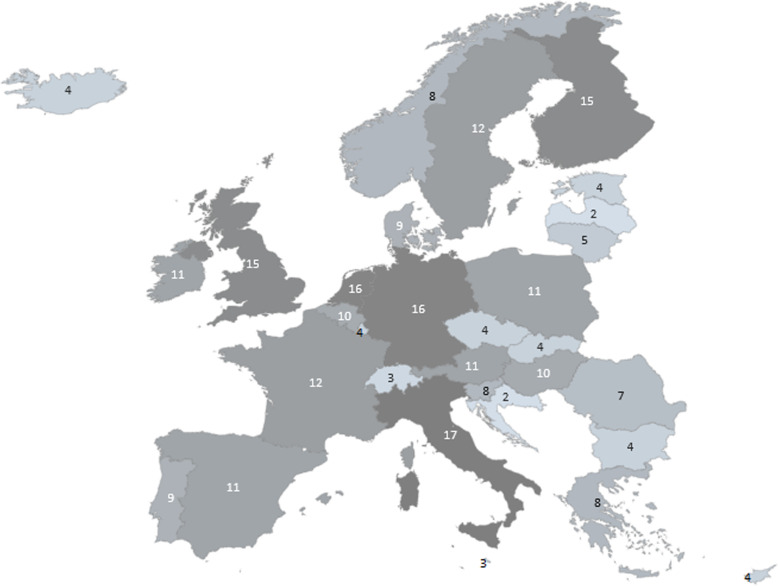
Geographical presentation of participation in selected SHH networks & projects. numbers represent the number of projects a country participated in

It appears that MS participation in population health projects follows a similar pattern as MS participation in health systems/services (see Figs. 2 and 3, respectively). Overall participation in health systems/services projects is less than MS participation in projects in population health.

## Discussion

International collaborations and comparisons of population health and care information can drive the exchange of best practices and learning from our neighbours [13, 14], support setting national priorities and improve the health (information) system. The domain of population health and health systems is vast and much differentiated, as is the European health data and information landscape. Many national and international agencies, institutes, programs, projects and committees play a role in harmonizing, collecting and disseminating existing and new health data and indicators as fuel for health research and health policy support.

In this project retrieval and review we identified policy supporting health information projects and networks and explored ways to improve their legacy and sustainability. At the national level, various international networks, e.g. Euro-Peristat, EUROCARE and Eurosafe (see Table 2), have contributed importantly to setting and keeping national policy agendas and priorities by pointing at undesirable health differences [15]. The work by the EHLEIS projects in providing comparisons of healthy life expectancies across the EU has established an important core indicator for comparing health among EU Member States. The ECHI projects have successfully developed a set of indicators providing a snapshot of public health and care and an evidence-base for policy makers. Our review shows that several projects with a wider MS coverage have existed more than ten years. They have regular and rather complete data collections, substantial output in the form of several articles and/or reports, and often recognizable policy influence. From the results, policy-supporting projects and networks in the area of health system performance assessment (HSPA) appear scarce [16]. However, the past years did show an increase in HSPA initiatives by the larger institutions in the European region, such as the OECD, the European Observatory on Health Systems and Policies, the WHO Regional Office for Europe and the European Commission.

### Outputs

The CORDIS and Health Programmes databases are valuable sources of information. This is especially true because many original project websites have disappeared (e.g., ECHIM.org) and/or no longer provide access to project databases (e.g., ENHIS2, IBD, Health Data Navigator). However, these databases are not always complete, are not easy to search and contain many dead links. The titles in the ‘outputs’ tab of the Health Programmes database are not self-explanatory (making it difficult to see what the underlying documentation is about), content outputs and administrative output are displayed together and it is not clear why some deliverables are accessible and other are locked (e.g. I2SARE and JAMIE final reports). Easy access to these reports is especially important as policy supporting information is probably shared more via reports than via scientific articles. The latter may be a more important output for the Framework Programmes than for the Health Programmes. Difficulties finding project information has also been pointed out by Zander and Busse in their analysis as part of the EUROREACH project; they recommend stronger action on dissemination of outputs to the research community, policy makers and funders [16]. Lost output may lead to duplication in research and project topics, having to reinvent the wheel and inefficient resource allocation. Therefore, we here put forward the suggestion to EU-funding agencies to request an archived website as a deliverable for EU-funded projects, to be stored by the funding agencies in an easily accessible and searchable database.

### Information inequalities

Health information inequalities may be linked to multiple causes: the lack of accessibility and availability of data within a country to be able to monitor and evaluate; low quality of the existing data; lack of capacity and resource to analyze and work with data and translate it to policy relevant evidence; and finally low collaboration at international/EU level through participation in comparative research. From the information retrieved in this study we were able to look at this last aspect and note that some countries appear to participate in projects substantially less than others. Part of these projects aim to cover the full EU and therefore also benefit health information development in non-actively participating countries: e.g. ECHI and Euro-Healthy. Even when not actively engaged in the project, a country may (eventually) take part in a network after first developmental work by part of the countries (e.g., Eurosafe, Euro-Peristat, EUROMOMO). Also, participation in our index project may not be recorded accurately in the project database or it may be different from participation in related projects. Nonetheless, it gives an indication of country participation in EU funded projects. Eastern European countries showed lower participation. Many of these joined the EU in 2004 and some in 2007, but they may have already been eligible for funding most of the time span in scope. Possibly, their health information infrastructure has not yet developed as the other countries, and national institutes that can participate in or lead European networks have not (yet) been available so much. Although a full appreciation of this inequality in participation requires a more thorough evaluation and explanation, we speculate that lack of national funding opportunities for continuation, lack of awareness of funding instruments, lack of available capacity, data collecting entities and expertise, and lack of awareness of existing or previous networks, may be among the causative factors.

Collecting and providing a health information base for policy making is a complex issue, which is addressed differently in different countries. National priorities in health and care may drive different data collections, especially considering that some types of data collections (cohort studies, health examination surveys, disease registries) are very costly and labor-intensive. Furthermore, countries may not have a good overview of all health data collected and parties involved, or a clear information strategy for new data collections. Similarly, overviews of national experts that participate in international projects and international efforts at developing and harmonizing indicators, classifications and interoperability issues may be lacking. For optimal benefit, it is therefore crucial to increase country participation in EU projects and networks. Many new initiatives have specific attention for capacity building across countries, e.g. PHIRI [8] and the new Health Expectancy Network (HEX.net). The latter will specifically involve researchers from Eastern European countries as they were under-represented previously in EHLEIS and will address the observation that methodological advances are unevenly dispersed across Europe. Furthermore, setting up a system of National Nodes on Health Information, as suggested by InfAct and PHIRI, may contribute to a systematic EU-wide health information coordination and strategy, and reduce inequalities. National Nodes are developed as organizational entities linked to a national institution or governmental unit that bring together national stakeholders and discuss core issues in health information domains of national and international relevance for the country [7, 17].

It is also essential to ensure the sustainability of recent and existing projects and networks. Safeguarding their knowledge base and making it easily accessible, provides the opportunity for countries to build on the expertise that has already been developed in Europe.

### Sustainability issues and solutions

Several projects and networks are experiencing problems to continue their work in a sustainable manner. For example, the JAMIE project aimed to improve the Injury Database (IDB) system and transfer it to the European Statistical System, as part of the set of public health statistics [18], but these efforts were unsuccessful. DG SANTE no longer hosts the database. Nevertheless, the Eurosafe network still produces reports based on the collected data, but notices that “the number of data suppliers increased during the JAMIE project till 2013 but dropped in 2014 after the termination of EU co-funding. However, a stable core of 18 countries remained, which are still collecting and sharing IDB-data, despite lack of any EU-funding” [19]. Similarly, the EHES network’s website currently reads: “At the moment, activities of the EHES Coordinating Centre are limited due to lack of sustainable funding” [20]. Finally, the ECHI set, produced and maintained by 4 consecutive projects (1998–2012) currently has no governance or funding and is in need of a sustainable solution for its maintenance and improvement [21].

A few networks have found sustainable solutions by having their outcomes (data and indicator definitions) incorporated in Eurostat’s data collections or available through the Joint Research Centre (JRC), e.g. the EUROCAT network and the data from national cancer registries. Others have mainly built further on national support, such as the EUROMOMO project, a network that recently proved very relevant for estimating excess mortality during the COVID-19 pandemic. Some projects, e.g. EUROCARE projects on cancer survival, sustainably provide important healthcare quality indicators for all EU Member States. The EHLEIS network is turning towards the mechanism of COST action (CA) to keep the network alive (HEXnet), but this may not be a solution for data-collecting networks. The Euro-DEN network continued its work in liaison with the European Monitoring Centre for Drugs and Drug Addiction (EMCDDA; which participates in the network’s Steering Committee) after initial EU funding and has contributed to the European Drug Report for the last 6 years, as well as to ad hoc EMCDDA publications including Trendspotter studies and various risk assessments and scientific publications. The Reitox network is functioning as national drug observatories because of the Regulation governing the EMCDDA’s work. Regulation is an effective, albeit strong instrument, to achieve comparable data collections with large coverage, hence also reducing health information inequalities.

When further reviewing the set of projects and networks, it appears from our selection that networks coordinated by experts from national public health institutes or existing national registries and institutionalized data collections may provide a more solid base for international collaboration. For example, the Euro-Peristat network coordinated by the Institut national de la santé et de la recherche médicale (INSERM), and the EHES network coordinated by the Finnish Institute for Health and Welfare (THL). But, additional EU-support in excess of co-funding would still be needed for many networks to keep core business going and/or to expand some networks to full EU-wide coverage. Such support could assist capacity building and improvement of data availability and quality in non-committing MS, thereby also combating health information inequalities. Many networks were formed by bottom-up initiatives from a limited number of MS and were not guided by a systematic European health information agenda or subject priority list. Stimulating a strong collaboration and agenda setting by collaborating national public health institutes and/or national disease registries may be a valuable mechanism to arrive at a stronger EU health information system. The EC may further support international data harmonization and collection by supporting the improvement of interoperability, cross-border data exchange and the use of standards and classifications. Intensifying the collaboration with WHO and OECD in the area of health information is essential in this. In a summary of research on performance indicators and benchmarking for health system performance assessment, it was concluded that a clearinghouse function and systematic sharing of research findings with policy makers would be necessary, besides the need for further indicator development and more and better data [22]. Although we see some progress these conclusions still hold.

Currently, there is no institution at European level that digests the available and new research outcomes, data and evidence into health policy recommendations at the national and EU-level. Also, links between institutions within each country and between projects are not complete or continuous. Knowledge about the national health information systems is fragmented, but reveals considerable inequality [4]. The aim of working towards a European health information infrastructure, as taken up by InfAct in the form of the Distributed Infrastructure on Population Health (DIPoH) functioning as a European Research Infrastructure Consortium (ERIC) [23], is therefore a logical step. At this point in time it is uncertain what the trajectory will be for the establishment of the Infrastructure. As a first step to improve both the national and international coordination of health information, InfAct has initiated the development of and support for National Nodes on Health Information. Its progress can be monitored via the Health Information Portal.

### Limitations

We will almost certainly have missed some projects and networks, for example because the search terms did not reach them, because they appeared after our search was performed, or because we misjudged in applying the inclusion criteria. Also, we could not always find all the information needed.

## Conclusion

Many EU projects and networks have worked on comparable data and indicators in the area of public health and health systems performance. It is a challenge for networks at the international level to find funding to continue their activities, including exchange among partners, collecting data and publishing reports and scientific articles. An important problem in this regard is the lack of an infrastructure to embed all this information in and prevent the loss of previously gathered knowledge, expertise and data that can be further built upon. Project outputs are difficult to access, and despite efforts by the EC collecting information in their databases, a wealth of information is lost each time a website is taken offline. We recommend that the EC prepare a mechanism for archiving these websites, as well as systematically evaluating the outcomes of the funded projects.

It is unclear to what extent health information inequalities exist in the European Region, and to what extent limited capacity to make decisions based on (contextualized) data within weaker information systems translates into less effective policy and disparities in health (care). More research is needed on this.

Finally, high-quality health information research networks are extremely important, but there is also the issue of political will that still needs to be addressed. We recommend to further develop ways to exchange best practices and discuss common problems among the networks and with policymakers, via the Health Information Portal and National Nodes on Health Information. Consolidation of previous efforts in the form or a sustainable infrastructure is necessary.

## Supplementary Information

**Additional file 1.** This file contains the search terms used in this article.

## Data Availability

NA/Data sharing is not applicable to this article as no datasets were generated or analysed during the current study.

## Abbreviations

DG SANTE: Directorate General Health and Food Safety
DIPoH: Distributed Infrastructure on Population Health
HIP: Health Information Portal
InfAct: Information to Act!, Joint Action on Health Information
PH: Population health
HSS: Health systems/services
