# Supplementary material for: EU health information progress: the harvest of policy supporting projects and networks
Source: Arch Public Health. 2022 Jan 30;80:47. doi: 10.1186/s13690-021-00772-4 (PMC8800830; doi:10.1186/s13690-021-00772-4)
Supplement: Supplementary file 1 — Additional file 1. This file contains the search terms used in this article. [file 13690_2021_772_MOESM1_ESM.docx]

# Additional file 1

**Search terms bibliographic databases (PubMed, Embase, Scopus)**

***PubMed search strategy:***

1. "health information"[ti] OR "health knowledge"[ti] OR "health data"[ti] OR "health registries"[ti] OR "health monitoring"[ti] OR "health indicator"[ti] OR "health indicators"[ti] OR echi[ti] OR "health policy"[ti] OR "public health"[ti] OR "population health"[ti] OR "national health"[ti] OR "health care"[ti] OR healthcare[ti] OR "health system"[ti] OR "health systems"[ti] OR "health services "[ti] OR "health surveillance"[ti] OR "health monitoring"[ti] OR "health reporting"[ti] OR "population health assessment"[tiab] OR "health system assessment"[tiab] OR "health system performance"[tiab] OR "health services performance"[tiab] [229.608]

2. "health information"[ot] OR "health knowledge"[ot] OR "health data"[ot] OR "health registries"[ot] OR "health monitoring"[ot] OR "health indicators"[ot] OR echi[ot] OR "health policy"[ot] OR "public health"[ot] OR "population health"[ot] OR "national health"[ot] OR "health care"[ot] OR healthcare[ot] OR "health system"[ot] OR "health systems"[ot] OR "health services performance"[ot] OR "health surveillance"[ot] OR "health monitoring"[ot] OR "health reporting"[ot] [84.755]

3. "public health informatics"[mh] OR "public health surveillance"[mh] OR "health information management"[mh] OR "public health"[mh] OR "population health"[mh] OR "population surveillance/methods"[mh] OR "health status indicators"[mj] OR "health surveys/methods"[mh] OR "delivery of health care"[mh:noexp] OR "health care surveys"[mh:noexp] OR "quality indicators, health care"[mh] [7.323.855]

4. #1 OR #2 OR #3 [7.458.901]

5. "health information base"[ti] OR "health information system"[ti] OR "health information systems"[ti] OR "health information tool"[ti] OR "health information tools"[ti] OR "health information base"[ot] OR "health information system"[ot] OR "health information systems"[ot] OR "health information tool"[ot] OR "health information tools"[ot] [1.035]

6."health information system"[ti] OR "health care information system"[ti] OR "healthcare information system"[ti] OR "health information systems"[ti] OR "health care information systems"[ti] OR "healthcare information systems"[ti] OR "information tool"[ti] OR "information tools"[ti] OR "data infrastructure"[ti] OR "indicator system"[ti] OR "indicator systems"[ti] OR "health monitoring system"[ti] OR "health monitoring systems"[ti] OR "health information system"[ot] OR "health care information system"[ot] OR "healthcare information system"[ot] OR "health information systems"[ot] OR "health care information systems"[ot] OR "healthcare information systems"[ot] OR "information tool"[ot] OR "information tools"[ot] OR "data infrastructure"[ot] OR "indicator system"[ot] OR "indicator systems"[ot] OR "health monitoring system"[ot] OR "health monitoring systems"[ot][1.600]

7. ("data collection"[ti] OR "data reporting"[ti] OR "data source"[ti] OR "data sources"[ti]) AND (tool[ti] OR tools[ti] OR base[ti] OR system[ti] OR systems[ti] OR project[ti] OR projects[ti] OR "data infrastructure"[ti]) OR ("data collection"[ot] OR "data reporting"[ot] OR "data source"[ot] OR "data sources"[ot]) AND (tool[ot] OR tools[ot] OR base[ot] OR system[ot] OR systems[ot] OR project[ot] OR projects[ot] OR "data infrastructure"[ot]) [314]

8. "expert network"[tiab] OR "expert networks"[tiab] OR "data collecting network"[tiab] OR "data collecting networks"[tiab] OR "expert network"[ot] OR "expert networks"[ot] OR "data collecting network"[ot] OR "data collecting networks"[ot] [71]

9. ("health information systems"[mh] OR "health information exchange"[mh] OR (("data collection/standards"[mh:noexp] OR "registries/standards"[mh:noexp] OR "health surveys/standards"[mh:noexp] OR "population surveillance/methods"[mh] OR "population surveillance/standards"[mh] OR "public health surveillance/methods"[mh]) AND ("international cooperation"[mh] OR "international classification of diseases"[mh] OR "interinstitutional relations"[mh]))) AND (eu[ti] OR europe[ti] OR european[ti] OR eu[ot] OR europe[ot] OR european[ot] OR "europe"[mj:noexp] OR "european union"[mj:noexp]) [175]

10. #4 AND (#5 OR #6 OR #7 OR #8 OR #9) [1.925]

11. project[tiab] OR projects[tiab] OR project's[tiab] OR organizations[ti] OR project[ot] OR projects[ot] OR project's[ot] OR organizations[ot] [197.721]

12. "joint action"[tiab] OR (("international cooperation"[mh] OR "interinstitutional relations"[mh]) AND (eu[ti] OR europe[ti] OR european[ti] OR eu[ot] OR europe[ot] OR european[ot] OR "europe"[mj:noexp] OR "european union"[mj:noexp]) AND (health[ti] OR healthcare[ti] OR care[ti]))

[3.064]

13. #4 AND #11 AND #12 [204]

14. (network*[ti] OR project*[ti] OR indicator*[ti]) AND (health*[ti] OR healthcare[ti] OR care[ti] OR incidence[ti]) AND (eu[ti] OR europ*[ti]) [521]

15. europe[tiab] OR european[tiab] OR eu[tiab] OR efta[tiab] OR "europe"[mh] OR "european union"[mh] OR europe[ot] OR european[ot] OR eu[ot] OR efta[ot] [1.514.889]

16. (#10 OR #13 OR #14) AND #15 [1.089]

17. #16 NOT ("africa"[mh] OR "asia"[mh] OR "americas"[mh] OR "australasia"[mh] OR "oceania"[mh] [1.044]

18. #17 NOT (infectious[ti] OR communicable[ti] OR rare[ti] OR "communicable diseases"[mh] OR "rare diseases"[mh]) [1.008]

19. #18 AND 2010:2019[dp] AND english[la] [446]

***Embase search strategy:***

1. 'health information':ti OR 'health knowledge':ti OR 'health data':ti OR 'health registries':ti OR 'health monitoring':ti OR 'health indicator*':ti OR echi:ti OR 'health policy':ti OR 'public health':ti OR 'population health':ti OR 'national health':ti OR 'health care':ti OR healthcare:ti OR 'health system*':ti OR 'health services':ti OR 'health surveillance':ti OR 'health monitoring':ti OR 'health reporting':ti OR 'population health assessment':ti,ab OR 'health system assessment':ti,ab OR 'health system performance':ti,ab OR 'health services performance':ti,ab [258.441]

2. 'medical informatics'/de OR 'medical information system'/de OR 'public health'/de OR 'population health'/de OR 'health status indicator'/mj OR 'health survey'/mj OR 'health care delivery '/de OR 'health care'/de OR 'health care surveys'/de OR 'quality indicators'/de [496.163]

3. #1 OR #2 [661.309]

4. 'health information base':ti OR 'health information system*':ti OR 'health information tool*':ti [872]

5. 'health information system*':ti OR 'healthcare information system*':ti OR 'information tool*':ti OR 'data infrastructure':ti OR 'indicator system*':ti OR 'health monitoring system*':ti [1.417]

6. ('data collection':ti OR 'data reporting':ti OR 'data source*':ti) AND (tool:ti OR tools:ti OR base:ti OR system:ti OR systems:ti OR project:ti OR projects:ti OR 'data infrastructure':ti) [622]

7. 'expert network*':ti,ab OR 'data collecting network*':ti,ab [107]

8. ('medical information system'/de OR (('data collection method'/de OR 'register'/de OR 'health survey'/de) AND ('international cooperation'/de OR 'international classification of diseases'/de))) AND (eu:ti OR europe:ti OR european:ti OR 'europe'/de OR 'european union'/de) [591]

9. #3 AND (#4 OR #5 OR #6 OR #7 OR #8) [1.774]

10. project:ti,ab OR projects:ti,ab OR 'project?s':ti,ab OR organizations:ti [258.754]

11. 'joint action':ti,ab OR ('international cooperation'/de AND (eu:ti OR europe:ti OR european:ti OR 'europe'/de OR 'european union'/de) AND (health:ti OR healthcare:ti OR care:ti)) [2.177]

12. #3 AND #10 AND #11 [72]

13. (network*:ti OR project*:ti OR indicator*:ti) AND (health*:ti OR healthcare:ti OR care:ti OR incidence:ti) AND (eu:ti OR europ*:ti) [703]

14. europe:ti,ab OR european:ti,ab OR eu:ti,ab OR efta:ti,ab OR 'europe'/de OR 'european union'/de [530.677]

15. (#9 OR #12 OR #13) AND #14 [1.239]

16. #15 NOT ('africa'/exp/de OR 'asia'/exp/de OR 'western hemisphere'/exp/de OR 'australia and new zealand'/exp/de) [1.088]

17. #16 NOT (infectious:ti OR communicable:ti OR rare:ti OR 'communicable disease'/exp/de OR 'rare disease'/de) [1.061]

18. #17 AND [2010-2019]/py AND english:la [517]

***Scopus search strategy:***

1. TITLE((health-information) OR (health-knowledge) OR (health-data) OR (health-registries) OR (health-monitoring) OR (health-indicator*) OR echi OR (health-policy) OR (public-health) OR (population-health) OR (national-health) OR (health-care) OR healthcare OR (health-system*) OR (health-services) OR (health-surveillance) OR (health-monitoring) OR (health-reporting)) OR TITLE-ABS((population-health-assessment) OR (health-system-assessment) OR (health-system-performance) OR (health-services-performance)) [324.256]

2. KEY((public-health-informatics) OR (public-health-surveillance) OR (medical-informatics) OR (medical-information-system*) OR (health-information-management) OR (public-health) OR (population-health) OR (population-surveillance) OR (health-status-indicator*) OR (health-survey*) OR (health-care-delivery) OR (delivery-of-health-care) OR (health-care) OR (health-care-survey*) OR (quality-indicators)) [1.776.772]

3. #1 OR #2 [1.891.693]

4. TITLE((health-information-base) OR (health-information-system*) OR (health-information-tool*)) [1.339]

5. TITLE((health-information-system*) OR (healthcare-information-system*) OR (information-tool*) OR (data-infrastructure) OR (indicato-system*) OR (health-monitoring-system*)) [4.083]

6. TITLE((data-collection) OR (data-reporting) OR (data-source*)) AND TITLE(tool* OR base OR system* OR project* OR (data-infrastructure)) [1.974]

7. TITLE-ABS((expert-network*) OR (data-collecting-network*)) [462]

8. KEY((medical-information-system*) OR (health-information-system*) OR (data-collecting-network*)) OR (KEY((data-collection-method*) OR regist* OR (health-survey*) OR (polulation-surveillance-methods) OR (public-health-surveillance-methods)) AND KEY((international-cooperation) OR (international-classification) OR (interinstitutional-relations)) AND TITLE(eu OR europ*)) [23.744]

9. #3 AND (#4 OR #5 OR #6 OR #7 OR #8) [25.772]

10. TITLE-ABS(project*) OR TITLE(organizations) [1.797.455]

11. TITLE-ABS(joint-action) OR (TITLE-ABS-KEY(international-cooperation) AND (TITLE(eu OR europe*) OR KEY(eu OR europe*)) AND TITLE(health OR healthcare OR care)) [6.129]

12. #3 AND #10 AND #11 [192]

13. TITLE(network* OR project* OR indicator*) AND TITLE(health* OR healthcare OR care OR incidence) AND TITLE(eu OR europe*) [702]

14. TITLE-ABS-KEY(eu OR europe* OR efta) [1.428.787]

15. (#9 OR #12 OR #13) AND #14 [2.049]

16. KEY(africa* OR asia* OR (western-hemisphere) OR australia OR (new-zealand) OR america*) [1.223.535]

17. TITLE(infectious OR infections OR communicable OR rare) OR TITLE(infectious OR infections OR communicable OR rare) [695.118]

18. #15 AND NOT (#16 OR #17) [1.879]

19. LANGUAGE(english) and PUBYEAR AFT 2009 [24.505.252]

20. #18 AND #19 [887]

21. TITLE(network* OR system* OR project* OR program* OR source* OR infrastructure* OR collaboration OR comparison* OR indicator* OR performance OR europ* OR initiated) [6.965.450]

22. #20 AND #21 [612]

**Search terms google**

***Google search strategy***

intitle:"health information" network (eu OR europe OR european)

intitle:"health information" network (transnational OR "cross national" OR cooperation OR countries OR international)

intitle:"health information" project (eu OR europe OR european)

intitle:"health information" project (transnational OR "cross national" OR cooperation OR countries OR international)

intitle:network "health information" (eu OR europe OR european)

intitle:network "health information" (transnational OR "cross national" OR cooperation OR countries OR international)

intitle:"health indicator" network (eu OR europe OR european)

intitle:"health indicator" network (transnational OR OR "cross national" cooperation OR countries OR international)

intitle:project "health indicator" (EU OR europe OR european)

intitle:project "health indicator" (transnational OR OR "cross national" cooperation OR countries OR international)

Variations to this theme were also included.

**Search terms EU project databases**

***CHAFEA health programmes database:***

July 2019, updated April 2020

- Advanced search 1:
  - Countries: EU and EFTA countries
  - Project year: ≥2010
  - Portfolio: “Data collection, Health indicators, Health reports, Indicators and data”

[n=8]

- Advanced search 2:
  - Countries: EU and EFTA countries
  - Project year: ≥2010
  - Key words: health information, check box for “environmental factor”, “lifestyle”, “methods”, “non-communicable diseases”, “socioeconomic factors” -> annex

[n=216]

[search 1+2: n=217 unique results]

- All projects in ‘health’ and include project call "Health Information (Hi 2003)" and include project call "Health Information (Hi 2004)" and include project call "Health Information (Hi 2005)" and include project call "Health Information (Hi 2006)" and include project call "Health Information (Hi 2007)"

[n=137]

***Cordis***

July 2019, updated April 2020

- Search 1:
  - Search: “health information” AND (population OR public)
  - Collection: Projects, Project Deliverables, Project Publications
  - Domain of Application: Health
  - Programme: FP7 FP6 H2020
  - Language: English
  - Start date (From): 2010-01-01

(/result/relations/categories/collection/code='publication','deliverable' OR contenttype='project') AND (programme/code='H2020' OR programme/code='FP7' OR programme/code='FP6') AND applicationDomain/code='health' AND ('health information' AND ('population' OR 'public'))

[n=48]

- Search 2:
  - Search: (compar* OR benchmark*) AND (health AND (population OR public))
  - Collection: Projects, Project Deliverables, Project Publications
  - Domain of Application: Health
  - Programme: FP7 FP6 H2020
  - Language: English
  - Start date (From): 2010-01-01

(/result/relations/categories/collection/code='publication','deliverable' OR contenttype='project') AND (programme/code='H2020' OR programme/code='FP7' OR programme/code='FP6') AND applicationDomain/code='health' AND (('compar*' OR 'benchmark*') AND ('health' AND ('population' OR 'public')))

[n=392]

[search 1+2: n=411 unique results]
